# Highly Efficient Planar Hot Electron Emitters Based on Ultrathin Pyrolyzed Polymer Films

**DOI:** 10.1021/acsami.4c19809

**Published:** 2025-05-30

**Authors:** Florian Herdl, Natalie Galfe, Sebastian Klenk, Michael Dillig, Silke Boche, Michael Bachmann, Andreas Schels, Simon Edler, Florian Dams, Andreas Pahlke, Georg S. Duesberg

**Affiliations:** † Institute of Physics & Center for Integrated Sensor Systems (SENS), 26576 University of the Bundeswehr Munich, Neubiberg 85579, Germany; ‡ 529691 KETEK GmbH, Munich 81737, Germany

**Keywords:** hot electron emission, Fowler−Nordheim tunneling, electron injection, pyrolyzed polymer films, pyrolysis, conductive carbon films, graphenic carbon

## Abstract

Miniaturized integrated hot electron emitters are highly sought after for application in chemical analytics and field-applicable systems. Here, we present the use of ultrathin pyrolyzed polymer films (PPFs) as the gate electrode, enabling the fabrication of highly efficient planar hot electron emitters (PHEEs). The thickness of the PPF was observed to be roughly 1 nm across a full 4” wafer, approaching the monolayer limit. Conductivities of up to 3.5 × 10^4^ S/m at pyrolysis temperatures of only 900 °C were measured, representing a 2-fold increase compared to bulk values. This renders an easily accessible 2D material with high electron transparency. Thus, the PHEE exhibits very high transfer ratios of up to 31% and proves to be stable at high pressures over an extended period of time. Furthermore, the straightforward integration route of the PPF presented here comprises only two steps: photolithography and subsequent pyrolysis. The fabricated devices exhibit high uniformity in performance, with a transfer ratio standard deviation of 2.9% across a single wafer. Ultimately, the devices were fabricated exclusively with silicon dioxide on silicon in combination with carbon, which represents a sustainable fabrication approach with inert materials. It has been demonstrated that the PHEE can also operate in both nitrogen and air, illustrating the utility of these emitters for gas ionization and sensing.

## Graphical abstract

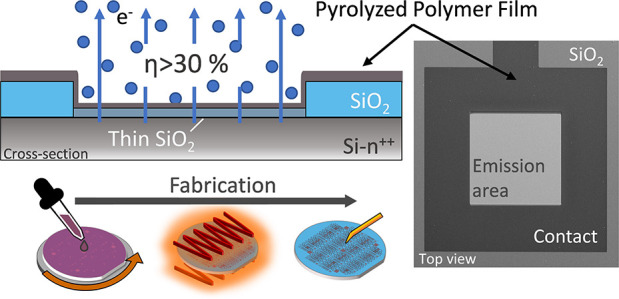

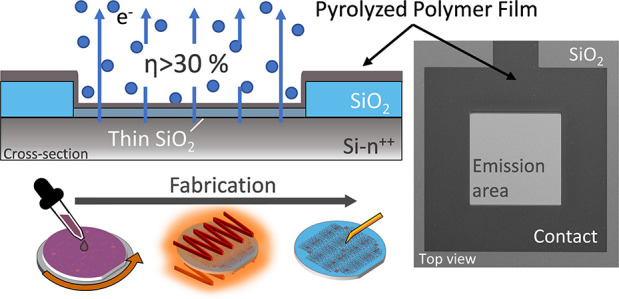

## Introduction

Electron emission technologies are nowadays widely utilized in many key technological applications, such as high-resolution electron microscopy, electron beam lithography, and X-ray generation. In the majority of cases, thermionic emitters, field emitters, or hybrid Schottky emitters are employed. To achieve sufficient lifetime for industrial use, they are typically operated under high vacuum. However, with the growing interest in miniaturization and the integration of devices into on-chip and field-applicable systems, there is a high demand for an alternative that is less susceptible to the associated challenges, such as operation under low vacuum. Furthermore, applications such as ionization sources or electron capture detectors could benefit from a miniaturized source of free electrons at atmospheric pressure.^1,2^ In particular, a scalable electron emitter for atmospheric operation could significantly enhance on-chip chemical analytics. Planar hot electron emitters (PHEEs) based on metal–oxide–semiconductor (MOS) heterostructures are promising alternatives to the currently established electron emitters. They have the potential to expand the scope of applications of free electrons.

The operating principle of the PHEE is based on the tunneling process from the semiconductor substrate to the conduction band of the oxide. Figure 1a illustrates the energy band diagram of the PHEE. By applying a bias to the conductive gate layer, a high electric field (approximately 1 V/nm) is generated across the oxide, resulting in the formation of a near-triangular barrier at the semiconductor/oxide interface. A tunneling current through the triangular barrier occurs (*I*_FN_), which can be approximated by the Fowler–Nordheim equation.^3^ After the tunneling process, the electrons gain energy as a result of the high electric field in the insulator, thus causing them to heat up. However, due to inelastic scattering events, the electrons occasionally lose energy and the energy distribution of the electrons spreads.^4^ The utilization of thin layers and an appropriate combination of materials ultimately facilitates the transmission of electrons through the gate material into the surrounding medium (emission current; *I*_emi_). The transfer ratio η, which is frequently employed as the figure of merit for PHEEs, is calculated by taking the ratio of the emission current to the current from the silicon substrate.

**Figure 1:**
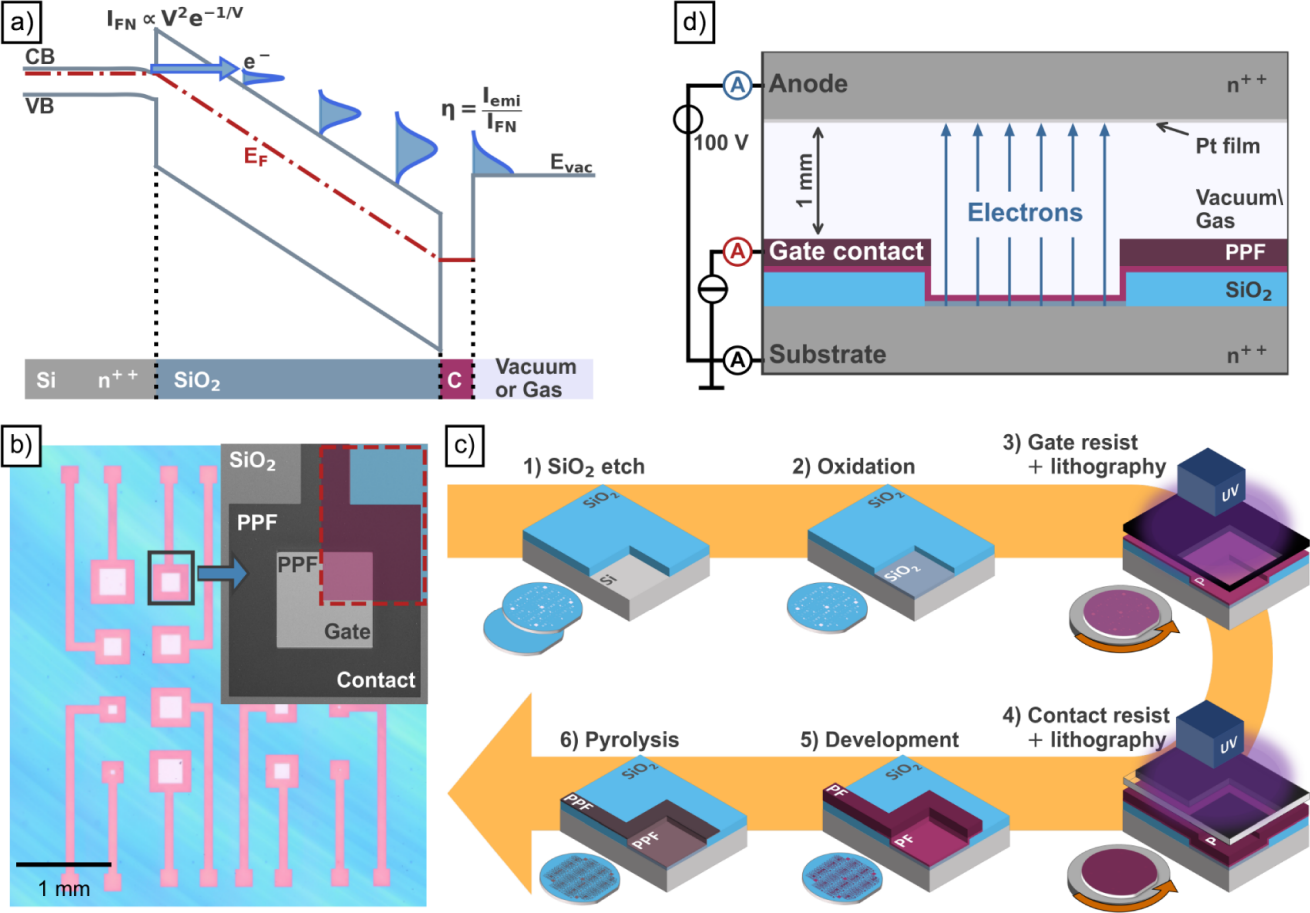
(a) Schematic energy band diagram, including the valence band (VB), conduction band (CB), and Fermi energy (*E*_F_), of a planar hot electron emitter (PHEE) consisting of silicon (Si), silicon dioxide (SiO_2_), and a carbon film (C) facing the vacuum or gas environment. The stack is biased by a gate voltage, enabling a tunneling current at the Si/SiO_2_ interface. After the spreading of the energy distribution due to scattering in the SiO_2_ and gate film, electrons that surpass the vacuum energy (*E*_vac_) can emit from the surface. (b) Micrograph of a PHEE array with an SEM inset of one emission site. The area shaded in red in the inset corresponds to the part of the PHEE that is used in Figure 1c to explain the fabrication steps in a cross-sectional representation. Starting from a highly n-doped Si/SiO_2_ 4” wafer, the emission sites are etched into the SiO_2_ (1) and subsequently oxidized again to form a 13 nm tunneling oxide (2). The gate resist (3) and the contact resist (4) are subsequently applied by spin coating and exposed by UV light with the respective lithography masks. After a joint development step (5), the remaining polymer film (PF) is pyrolyzed (6) to PPF. (d) Schematic measurement setup for electrical characterization of the PHEE. It was operated in constant gate current mode, with the substrate, gate, and anode currents being monitored.

One noteworthy advantage over conventional electron emitters that all PHEEs share is their exceptional performance at poor vacuum and even atmospheric pressure levels, which can be attributed to the buried tunneling barrier and high electric field within the solid. This makes them promising candidates for environments in which other electron emitters cannot operate. It has been demonstrated that the PHEE can be utilized in an electron lithography system,^5^ a scanning electron microscope under poor vacuum^6^ and as an ionization source under atmospheric pressure in an ion mobility spectrometer.^7^ However, the suitability of previous PHEEs for these applications is limited due to the low efficiency, complex gate fabrication, and poor reproducibility of the latter.

PHEEs based on the MOS heterostructures have been first described over 60 years ago by Mead^8^ and extensively discussed since then.^9–17^ The initial MOS structures that demonstrated electron emission were developed using metal films such as aluminum as the gate layer. However, despite the use of very thin metal sheets of down to 6 nm, the transfer ratio could not overcome 0.7%.^4^ The development of graphenic thin carbon layers has recently attracted attention in the field due to their potential use as highly conductive and electron transmissive gate layers.^14,15^ Murakami et al. achieved the most efficient PHEE so far, with a maximum transfer ratio above 48% by using ultrathin pyrolytic carbon films of only a few nanometers in thickness.^18^ It was possible to reach such high transfer ratios by depositing the gate layer at temperatures as low as 900 °C, to reduce carbon diffusion into the insulator, while depositing a very thin and highly conductive layer.^19^ However, this deposition at such low temperatures and pressures is difficult to achieve and requires a two-zone furnace, since the activation energy for precursor decomposition must be expended.^20^ Furthermore, only a few individual devices have shown high performances and reproducible fabrication remains a challenge.

In this contribution, we report a highly efficient gate electrode for PHEEs based on ultrathin pyrolyzed polymer films (PPF). The devices exhibit high transfer ratios and long-term stability. The use of PPF combines a scalable fabrication process with performances similar to best-in-class graphenic carbons. We demonstrate reproducible and durable hot electron emitters with wafer-scale processing. Furthermore, only Si/SiO_2_ and PPF were employed in the fabrication, providing an instance of a semiconductor-carbon device devoid of additional materials, contributing to a sustainable approach for device fabrication. The herein presented PHEE has great potential for multiple integratable devices and will further enhance the scope of applications of free electrons.

## Experimental Methods

The PHEEs are manufactured using a highly scalable and integratable process that is compatible with the infrastructure of current semiconductor factories. In addition to the structured Si/SiO_2_ substrate, the PPF gate only requires a spin coater, lithography, and an annealing furnace, which are commonly present in most production lines. Furthermore, the implementation of carbon films on the wafer-scale has already been demonstrated.^21^ Figure 1b depicts an optical micrograph of a complete array of differently sized PHEEs, wherein the pale pink squares are the emission areas circumscribed by thicker PPF contacts in bright pink. The colored area situated in the upper right-hand corner of the scanning electron micrograph (SEM) inset denotes the section that is schematically visualized in the illustration of the fabrication process in Figure 1c. Starting with a highly n-doped 4” silicon wafer, a wet oxidization at 900 °C to an oxide thickness of about 300 nm is performed. Subsequently, windows are etched into the oxide by buffered hydrofluoric acid to define the emission areas (1). After a standard RCA cleaning step, a dry tunneling oxide of approximately 13 nm is grown in a rapid thermal processing furnace at 1000 °C (2). In the next step, the carbon gate material is applied (3). In contrast to the conventional deposition of pyrolytic carbon via a CVD process^19^ or the transfer of catalytically grown graphene onto the substrate,^22^ ultrathin PPF is employed here as the gate material.^23^ For this, AZ nLOF 2070 photoresist is diluted down to 5 wt % in AZ EBR (MicroChemicals GmbH, Ulm, Germany) and spin coated onto the substrate wafer with rotational speeds of up to 6000 rpm. Since the polymer is a negative-type resist, the emission sites can already be defined by a photolithography mask, rendering additional postdeposition patterning obsolete. The resist is cross-linked by exposure to UV light and a subsequent baking step at 120 °C for 60 s. For the contact material, nondiluted photoresist AZ nLOF 2070 is used to achieve thicker and therefore more conductive contact paths to the emission sites. This process enables the fabrication of devices without the use of metals, thereby contributing to a more sustainable approach to device fabrication. After exposure and baking of the contact material, both resists are simultaneously developed in AZ 2026 MIF. PPF is formed by pyrolysis of the structured resist in a vacuum furnace under constant inert gas flow. For better process control, the temperature is gradually increased by 5 K/min up to 500 °C with a constant flow of 200 sccm argon at 0.1 mbar. Then, the rate is doubled to 10 K/min until 900 °C is reached, where the sample is pyrolyzed for 60 min, converting the photoresist to a well-conductive PPF. Afterward, the wafer can be diced enabling a high throughput of PHEEs.

The emission characteristics were examined in a vacuum needle prober operating at a residual gas pressure of 10^–4^ mbar. Figure 1d provides a schematic of the measurement setup. The substrate is connected to ground potential through a backside contact, while the gate current is kept at a constant value. This permits the investigation of the emission behavior under constant stress, irrespective of any changes in the PHEE that may be caused by oxide-induced degradation.^24,25^ To measure the emitted electrons, a platinum-covered highly n-doped silicon anode is positioned approximately 1 mm from the emission site. It is biased at 100 V to prevent space charge effects and to facilitate the collection of all emitted electrons. The substrate, gate, and anode (emission, *I*_emi_) currents are measured by individual picoammeters.

## Results and Discussion

The gate material is the most crucial component of the hot electron emitter alongside the tunneling oxide, as the transfer ratio is primarily limited by scattering of electrons within the oxide/gate stack. Thus, the gate layer should exhibit a low thickness while also maintaining a high conductivity for homogeneous emission. Furthermore, it is imperative that the oxide layer is not adversely affected by the deposition of the gate layer.

Despite its simple fabrication scheme, PPF is a highly conductive film due to its predominantly sp^2^ hybridized orbital configuration. During pyrolysis, the elevated temperature causes the more volatile, noncarbonic components in the resist to evaporate, while allowing the carbon atoms to rearrange and form sp^2^ and sp^3^ hybridized bonds.^26–28^ This loss of material and restructuring by the formation of sp^2^ and sp^3^ bonds leads to a shrinkage of the film. Figure 2a shows an atomic force micrograph (AFM) of the thin gate material (left) and the thick contact material (right) before and after pyrolysis. The material shrinks during the pyrolysis from 9.7 nm down to 1.1 nm and from 395 to 63 nm in the case of the gate material and the contact material, respectively. Simultaneously, pyrolysis smoothens the material from a root-mean-square roughness of 2.04 nm down to 550 pm for the contact material. For the gate material, the difference is less significant, leading to a decrease from 675 pm down to 450 pm. This can be attributed to the already thin resist film on a rather smooth silicon dioxide substrate with an RMS roughness of approximately 200 pm.

**Figure 2:**
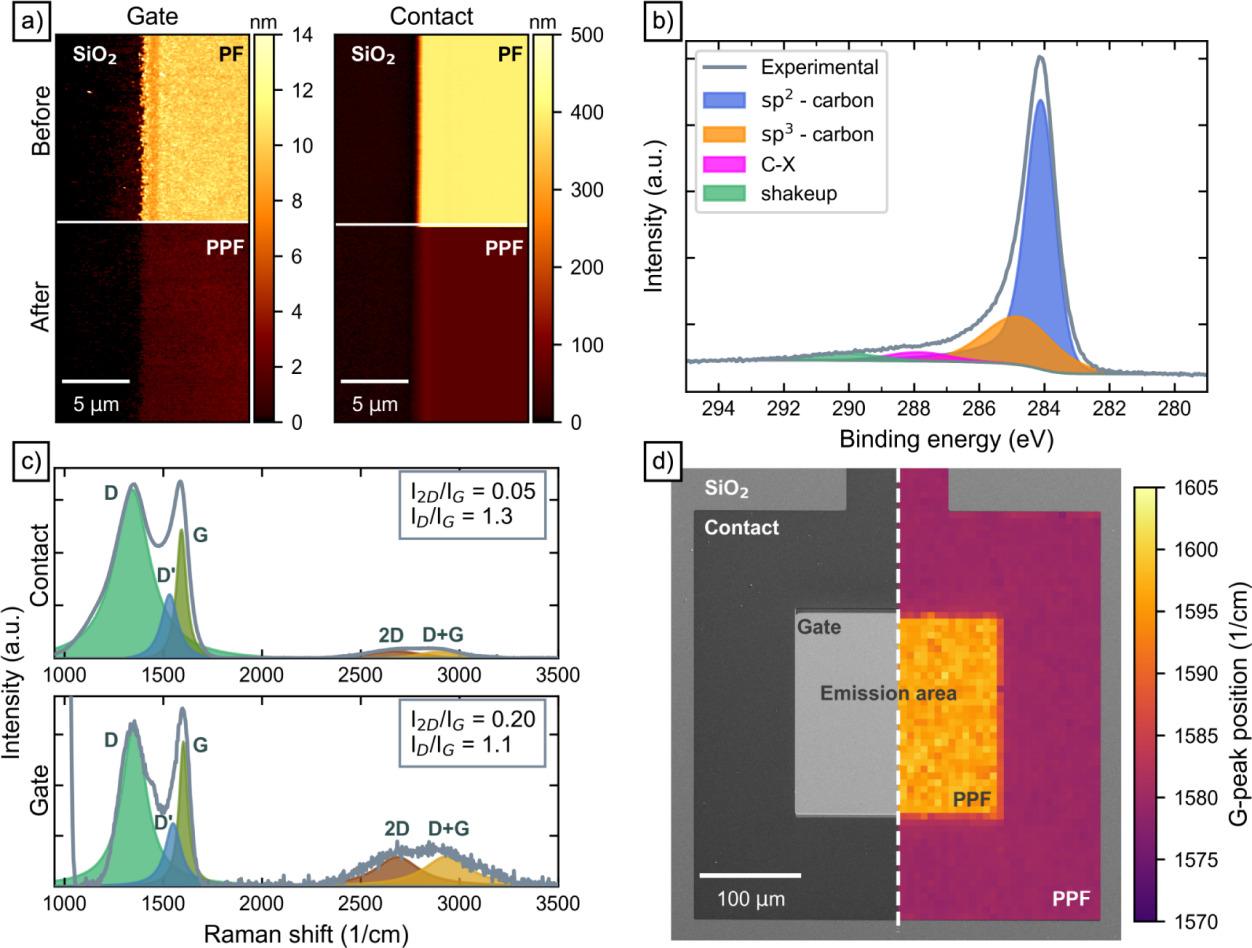
(a) Atomic force micrographs of the gate and contact polymer films conducted before (PF) and after pyrolysis (PPF), respectively. The edges were formed using photolithography. (b) X-ray photoelectron spectrum of the gate PPF. It shows mainly sp^2^ hybridized carbon bonds with only a small portion of sp^3^. (c) Raman spectra of the contact (upper) and gate (lower) PPF, respectively. Typical vibrational modes of PPF are indicated by Lorentzian-fits. The intensity ratios *I*_2D_/*I*_G_ and *I*_D_/*I*_G_ show a higher crystallinity for the thin gate PPF. (d) (Left) SEM of a planar hot electron emitter. The darker region is the 60 nm-thick PPF contact electrode surrounding the emission area composed of 1 nm-thick PPF in gray. (Right) Raman map of the G-peak position. The thicker PPF reveals a more amorphous material compared to the relatively nanocrystalline gate layer depicted in orange with an average G-peak position of 1602 cm^–1^.

An elemental analysis of the thin PPF was conducted using X-ray photoelectron spectroscopy (XPS), as shown in Figure 2b. The layer comprises predominantly sp^2^-hybridized carbon compounds with only a small proportion of sp^3^ hybridized bonds, hence indicating a high graphitization and consequently a high conductivity of the thin film. The bonds between the graphitized grains are partially sp^3^-hybridized, which explains their small signal observed in the XPS measurements. Another source for sp^3^-hybridized carbon is film defects. Feng et al. observed an increase in the sp^3^ signal for increasingly defective carbon films.^29^ Further carbon-related bonds are depicted by C–X, either caused by residues of the nonpyrolyzed photoresist film or pollutions from the surroundings. The low intensity of this peak implies an almost complete conversion of the film during pyrolysis.

Another effective method for obtaining additional structural data on the examined PPF is Raman spectroscopy. This technique is widely employed for the analysis of two-dimensional and thin film materials.^30–32^ The corresponding Raman spectra of the gate layer and the contact layer are depicted in Figure 2c, respectively. Carbon-based films show common vibrational modes between 1300 and 1650 cm^–1^ and 2000–3500 cm^–1^, giving insight into the film quality and layer thickness.^31,33^ The peaks can be attributed to the G-peak at 1603 cm^–1^, D-peak at 1353 cm^–1^ and D’-peak at 1551 cm^–1^ for the gate layer, which are in good agreement with the literature.^33,34^ Additionally, second-order vibrational modes such as the 2D-peak at 2687 cm^–1^ and D+G-peak at 2939 cm^–1^ can be seen at higher Raman shifts.^33^ The G-peak is related to the in-plane mode of sp^2^-hybridized carbon bonds for both rings and chains, whereas the D-peak originates from the breathing mode of sp^2^ hybridized carbon rings, neighboring defects or disorder of the graphene layer.^35^ Thus, the strong signal of the G-peak indicates a high amount of sp^2^ hybridization of the PPF, which is in good agreement with the previously discussed XPS characterization. The equally strong D-peak is typical for PPF and originates from the arbitrarily oriented and stacked polymer chains of the precursor, leading to a distorted configuration of the pyrolyzed film.^26^ The I­(2D)/I­(G) ratio depends on the graphitization and thickness of a graphenic carbon layer.^32^ An increase in the *I*_2D_/*I*_G_ ratio from 0.05 for the contact material to 0.2 for the gate material can be observed. An increase in the 2D peak can be caused either by a reduction in the number of graphene layers stacked on top of each other or by an increase in grain quality and size, resulting in an increase in intensity. Despite having only a few atomic layers of carbon present in the gate material, with a thickness of approximately 1 nm, a reduction in the number of stacked graphene layers compared to the thicker contact material is unlikely, since Jurkiewicz et al.^33^ have shown that stacked graphene clusters in PPF form at much higher temperatures (>1000 °C) than those presented in this work. They have argued that the low 2D-peak intensity at low pyrolysis temperatures could arise from the small lateral layer size of the graphene-like domains. Given that extremely thin films comprising just a few monolayers impede the formation of amorphous structures perpendicular to the surface, it is probable that they will give rise to more crystalline sheets, thereby increasing the intensity of the 2D-peak.

The improved crystallinity of ultrathin PPF layers is also evident in the position of the G-peak.^31^ This can be particularly discerned between the thicker contact areas and the ultrathin gate layer, both comprising PPF. Figure 2d depicts an SEM image of the PHEE structure on the left side, with an overlaid Raman map shown on the right. The darker region shown in the SEM micrograph is the PPF contact electrode of 63 nm thickness surrounding the emission area in gray, which is covered by the ultrathin PPF of 1.1 nm thickness. The Raman map of the G-peak position shows a clear difference between the gate layer and the contact layer, respectively. The thicker contact material in violet has a lower average Raman shift (1580 cm^–1^) for the G-peak, revealing a more amorphous material compared to the relatively nanocrystalline gate layer depicted in orange with an average G-peak position of 1602 cm^–1^.^31^

The process flow for the fabrication of the PPF-based PHEE is particularly well-suited for scalable applications, which makes it an attractive option for commercial use. To obtain a preliminary assessment of the process uniformity across the wafer, the sheet resistance is determined by examining 61 transfer length (TLM) structures equally distributed on a 4” wafer. The thin TLM channels (diluted resist) and the corresponding contacts (pure resist) were produced in the same manner as for the PHEE described above and consist of PPF. The channels exhibit a width of 100 μm and consist of seven segments with lengths ranging from 100 μm to 1.5 mm. It is important to note that all TLM dies on the wafer were functional resembling a yield of 100%. This is a huge advantage over numerous technologies using 2D materials such as CVD-grown graphene, which rely on wet chemical transfer.^22,36^ Nevertheless, we observe a considerable variation in sheet resistance of our PPF channel across the wafer. In Figure 3a, the distribution of the sheet resistances is depicted in a pseudocolor plot. The corresponding average contact resistance was found to be (2.0 ± 0.2) 10^6^ Ω μm, which aligns well with the geometry and resistivity of the lead contacts (see Supporting Information). The lowest value of the sheet resistance is observed near the middle of the wafer and gradually increases toward the edges. Despite the variation over the wafer, with a range of 1 order of magnitude, the resulting conductivities remain sufficiently high for the PHEE presented in this study.^37^ The origin of the inhomogeneity likely arises from the spin coating process, which naturally causes the resist to be thickest near the rotational axes. Note that the resist was applied manually in this case by using an Eppendorf pipet. This could lead to an off-centered resist puddle before spin coating, contributing to the inhomogeneity in thickness and ultimately to the sheet resistance. In an industrial context, such issues can be readily addressed through the use of automated spin coating systems or a spray coating process.

**Figure 3:**
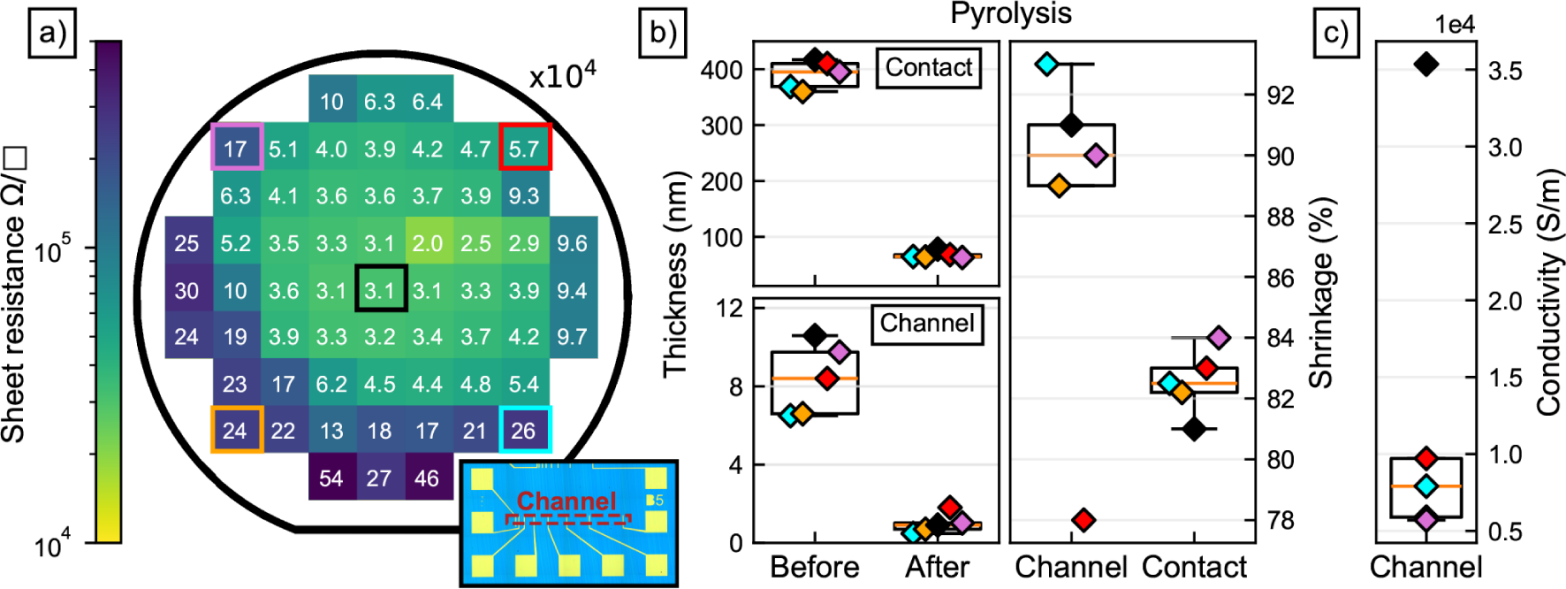
(a) Sheet resistance measurements conducted over a 4″ test wafer by the transfer length method (TLM). A micrograph of an exemplary TLM structure is shown in the inset, where the red marking indicates the pyrolyzed polymer film (PPF) channel and the yellow squares are the PPF contacts. The channel width is 100 μm. The colored spots are further characterized by atomic force microscope, whereby the thickness and the corresponding shrinkage are depicted in Figure 3b. From the sheet resistance and thickness values, a conductivity is calculated and shown in Figure 3c.

Thickness measurements of the gate and contact layers at five positions on the wafer were conducted. The five TLM dies framed in different colors in Figure 3a were measured with an AFM, similar to the micrographs shown in Figure 2a, and the results are shown in Figure 3b. The thicknesses of the thick contact and thin channel photoresist prior to the pyrolysis are (390 ± 22) nm and (8.4 ± 1.6) nm, respectively. As expected, the middle portion shows the thickest layer for both the channel and the contacts, with smaller thicknesses toward the edges. After pyrolysis, the distribution narrows and exhibits (0.98 ± 0.5) nm for the channel layer and (68 ± 5) nm for the thick contact layer. It is noteworthy that the initial thickness variations of the gate layer across the wafer are reduced to below measurement accuracy due to shrinkage during pyrolysis. PPF consists of multiple line-shaped crystallites originating from polymer chains interlaying with each other.^26^ Therefore, a random distribution in height could occur due to a randomly distributed and oriented crystallite formation during pyrolysis. Especially for the thin TLM channel, only a few additional overlapping crystallites have a drastic impact on the relative height, since the layer is already in the range of only a few monolayers of graphene.^38^

The total shrinkage can be calculated by the relative difference between the layer thickness before and after pyrolysis. It can be seen that this value is in good agreement with literature values of around 80%^23^ for the thicker contact layer and the resist used here. However, the thin material shrank up to 92%, likely due to the higher amount of solvent used to dilute the resist. Additionally, since the pyrolyzed layer already is in the range of a few monolayers only, less overlapping of polymer chains is possible, likely resulting in a flatter and more crystalline structure. This also aligns well with findings from the above-discussed Raman measurements.

With the thickness measurements from the five structures on the wafer and the sheet resistance, the corresponding conductivities can be calculated. The determined conductivities are depicted in Figure 3c. The edge TLM dies exhibit an average conductivity of (0.73 ± 0.16) 10^4^ S/m, which is in good agreement with the literature.^23^ However, on the central TLM die, an almost five times higher conductivity of (3.54 ± 0.21) 10^4^ S/m is observed. For pyrolysis, a cylindrical cold-wall reactor at low pressure was used, with carbon heaters at the top and bottom. Due to the low pressure, the main heat exchange occurs via thermal radiation with the reactor walls, causing a negative temperature gradient from the center to the edges of the wafer. Thus, the maximum temperature is achieved in the center of the wafer and cannot exceed the heater temperature set at 900 °C. Generally, higher temperatures lead to a higher graphitization of the layers and, therefore, higher conductivities, which explains the deviation of the center die to the edge dies.^23,26,33^ However, our thin layer even exhibits two times higher conductivities, compared to measurements conducted by Schreiber et al.^23^ Since we have already discussed the improvements of crystallinity due to decreasing layer thickness through Raman analysis and the thickness measurements, these relatively high conductivities at only 900 °C are in agreement with the above discussion. In conclusion, PPF resembles a highly conductive, yet very thin graphenic material, rendering it ideal for use as the gate material of a PHEE.

To investigate the IV characteristics of the fabricated emitters, the gate current is swept from 0.1 nA to 20 nA while the gate voltage is continuously monitored. For each current step, three consecutive measurements were performed to mitigate charging effects. Figure 4a shows the average of three consecutive gate current sweeps of a PPF-gated PHEE with an emission area of 300 μm × 300 μm in Millikan–Lauritsen representation.^39,40^ Other than the linearized FN plot, this representation features a readable *y*-axis (log­(*I*) vs 1/V), while maintaining a satisfactorily linearized representation of FN data for visualization. Further details are described elsewhere. To further improve readability, the *x*-axis has been inverted, and the labels of the reciprocal *x*-axis are directly presented as the gate voltage. The error bars represent the standard deviation over the sweeps at each current step. As expected,^3^ the nearly linear emission behavior in this representation identifies FN tunneling as the dominant conduction mechanism. A linear fit of the empirical data in Murphy–Good (MG) coordinates is anticipated to provide the most accurate theoretical fit for FN-type tunneling currents, since it also incorporates the image charge potential at the interface and, therefore, the lowering of the associated barrier.^41,42^ For this purpose, the correction term – *η/*6 is added to the classical FN coordinates (log­(*I*/*V* ^2–*η/*6^) vs 1/*V*) with $\eta ≅9.8\sqrt{eV/\phi }$ and ϕ being the work function. For further details, refer to the literature. MG fits with an assumed work function of 3.25 eV have been conducted,^3^ revealing a similar slope of (−180.09 ± 0.26) V and (−180.6 ± 1.9) V for both the gate and anode current. This similarity confirms that both originate from the same conduction path, ruling out the presence of leakage currents within the device. The transfer ratio of the PHEE, which is depicted in green, reveals constant electron transmission of (30.9 ± 0.5)%. This shows that PPF-gated PHEEs can compete with best-in-class PHEEs with pyrolytic carbon gates deposited by low-pressure chemical vapor deposition (LPCVD) (15%–48%).^19^

**Figure 4:**
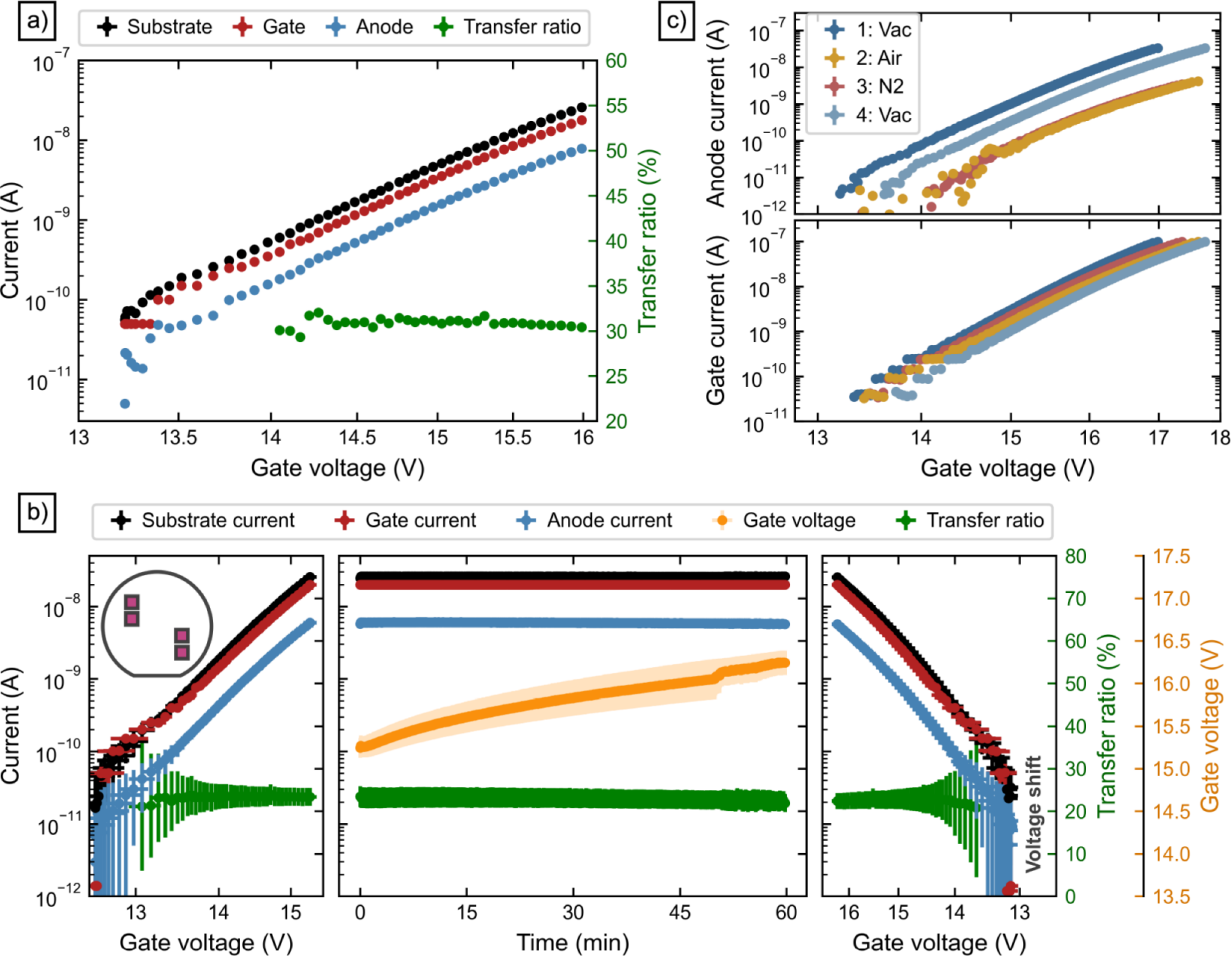
(a) IV characteristics curve of a planar hot electron emitter (PHEE) with a pyrolyzed polymer film (PPF) as the gate electrode. The curve shows high and stable transfer ratios of (30.9 ± 0.5)%. (b) Emission stability test of four PHEEs. The inset in the upper left corner roughly demonstrates the location of the examined PHEEs on the 4’’ wafer. The data show the mean values of all four combined PHEE measurements using standard deviation as the error bars. The first and last segments show *IV* characteristics in an adapted Millikan–Lauritsen representation before and after the stability test. In the middle segment, the stability test over 60 min is shown with the corresponding gate voltage propagation. (c) IV characteristics of the PHEE in different environments. It was first measured in vacuum (1), followed by nitrogen (2) and air (3). Subsequently, a second vacuum (4) measurement was conducted.

In a recent discussion on the mechanism behind PHEEs with high transfer ratios, Murakami et al.^18^ concluded that the fabrication of the gate layer should not affect the thin tunneling oxide, as this could induce scattering sites, e.g., diffused carbon atoms. The PPF process, without any further optimization, yields high conductivities at temperatures as low as 900 °C, thereby mitigating carbon diffusion into the tunneling oxide and contributing to the observed high transfer ratios. Moreover, we tentatively argue that carbon atoms that are already bonded to polymer chains prior to pyrolysis may exhibit a reduced propensity for diffusion into the oxide. To achieve high transfer ratios, it is also crucial for the gate material thickness to be ideally below the mean free path of electrons within the material, thus reducing scattering events during transmission through the gate.^18,19^ As demonstrated above, the minimum thickness achieved herein corresponds to only a few layers of carbon atoms. Therefore, the thickness of the gate layer is within the same order of magnitude as the mean free path, which ranges from 0.2 to 2 nm, for traversing electrons in graphite-based materials, dependent on the respective electron energy.^43,44^ Furthermore, due to its straightforward fabrication scheme, our PPF-based process is particularly suitable for temperature- and thickness-dependent studies, which will provide deeper insight into the underlying mechanisms of PHEEs.

In order to investigate the reproducibility and emission stability of the PPF-based PHEE, four different emission areas situated on different sites of a 4” wafer were examined via constant gate current measurements. Figure 4b presents a triptych representation of the conducted stability test. Two emission sites, located at the top left and two emission sites at the bottom right of the wafer were investigated, as illustrated in the inset in the upper left corner of the figure. The stability test comprises a constant gate current measurement at 20 nA for 60 min for each PHEE. Prior to and following the stability test, three consecutive gate current sweeps were conducted to investigate possible degradation effects of the tunneling mechanism. The data presented in Figure 4b illustrate the mean results of all combined PHEEs, using the total standard deviation per current step for the error bars. The *IV* characteristics are depicted in a modified Millikan–Lauritsen representation, as previously described. The stability test and IV characteristics demonstrate minimal fluctuations and high congruence across the four samples, indicating an exceptional homogeneity of the devices across the wafer. The prestability test IV characteristics exhibit a transfer ratio with a notably low standard deviation of (23.5 ± 2.9)%. Such high uniformity can be explained by the very well-controllable oxide thickness and its homogeneity combined with a low standard deviation of the gate layer thickness (0.98 ± 0.5) nm, as described above. However, in contrast to the aforementioned PHEE samples, the corresponding transfer ratio is lower. The two tests were conducted on two distinct wafer batches of PPF-based PHEEs. Given the high degree of consistency observed in the transfer ratio within a given wafer, it is reasonable to conclude that the cause is unlikely to be a random occurrence during pyrolysis, such as carbon diffusion. Instead, it is more probable that the variation is due to inherent differences in the resist used or variations in the lithography process. Similarly, it is possible that the cause is user error, given that, as previously stated, the lithography process has not yet been automated. Such issues could be rectified through the implementation of spray coating or automated spin coating systems. The precise cause of this discrepancy remains unknown, but due to the simple fabrication process, further studies on transfer ratio dependencies are planned. Ultimately, the transfer ratio yields high values, and the variation within a wafer seems remarkably low.

Since the measurement was carried out with a constant gate current, the gate voltage needs to be observed to determine any degradation during operation. In the stability test, the gate voltage has to increase by one volt, in order to sustain a gate current of 20 nA. A reproducible initial decrease in gate voltage can be observed during the first 30 s, after which the gate voltage increases steadily. This behavior is well known in the literature and can be attributed to the filling of existing and newly generated traps in the tunneling oxide.^24,25,45^ The injection of hot electrons into the gate results in the formation of positive charges (holes), which are trapped in close proximity to the gate within the oxide. In the event of a low electron fluence, these positive charges will predominate, leading to a voltage shift toward lower potentials. With an increase in electron fluence, negative charges trapped near the substrate will dominate, shielding the electric field needed for the tunneling process, and a positive voltage shift will be observed. This voltage shift is partially reversible, as charges in shallow traps will detrap after some time of nonoperation.^46^ Additionally, a persistent shift in voltage compared to the pristine device occurs, due to deep trap filling and surface trap generation in the early stages of operation. Further details are provided elsewhere. However, the lifetime of the device is limited by the ongoing generation of traps, ultimately forming a conductive path and causing the device to fail (breakdown).^47^ During long-term measurements, a breakdown of the oxide was observed after an injected charge of 0.43 C/cm^2^ (charge-to-breakdown, *Q*_BD_). Further details are described in the Supporting Information. Despite these changes in the tunneling oxide, the anode current and, consequently, the transfer ratio exhibit only slightly recognizable changes from (6.0 ± 0.6) nA (η: 23.2% ± 2.3%) at the beginning to (5.7 ± 0.5) nA (η: 22.2% ± 1.9%) at the end of the measurement. Therefore, the ongoing generation of new traps and the filling of already existing traps play a minor role in the scattering within the oxide, at most.

The voltage shift remains in the poststability *IV* characteristics, and the emission efficiency still shows only low fluctuations within the four PHEEs. A comparison of the MG slopes of all four PHEEs still demonstrates congruent values within the pre- or poststability test *IV* characteristics. However, the slopes increased from (−187.9 ± 2.2) V to (−172.3 ± 0.4) V over the course of the stability test. Negative charge trap filling and trap generation will not only compensate for the electric field, causing a parallel shift in gate voltage, but also alter the general tunneling behavior. This probably either originates from a change in the dielectric constant, height or shape of the tunneling barrier, or effective mass within the oxide.^3,42^

Those high stabilities, coupled with minimal degradation of the anode current during operation, offer great potential for the use of our PPF-based PHEE in a multitude of challenging environments. One such application is gas analytics, where the gate current can be easily controlled, while direct control of the active emission current is not straightforward due to ionization processes and gas transport influences.

In order to investigate the applicability of PHEEs in challenging environments, we conducted further analysis to examine their behavior in nitrogen and air. Accordingly, five consecutive gate current sweeps up to 100 nA were conducted in 1 atm nitrogen and in 1 atm air, respectively. Prior to and following the gas measurements, a vacuum measurement was conducted. Figure 4c depicts the anode and gate current in the top and bottom adapted Millikan-Lauritsen plots, respectively. The gate curves demonstrate a voltage shift of 0.7 V over the course of the test, again likely attributed to the filling of traps with negative charge carriers. Furthermore, the evolution of the curve remains unaltered, indicating that the presence of gas on the device surface does not affect its buried tunneling behavior. However, in contrast to the aforementioned vacuum measurements, it can be observed that the anode current is notably suppressed by a factor of 10 during the gas measurements, from a maximum of 33 nA in vacuum to 3.4 nA in nitrogen. This phenomenon has previously been observed in PHEEs measured at atmospheric pressure.^7,48^ It is likely that either adsorbed gas molecules on the surface alter the work function of the gate material, electron transport in the gas is constrained, or electrons get backscattered at the gas interface.^48^ However, this suppression of the anode current is fully reversible, as evidenced by the second vacuum measurement. The fact that this was possible without the need for temperature treatment or elongated vacuum annealing to promote the desorption rate suggests that the cause is not adsorption-based. In gas analytical applications, often low currents of only some 100 pA to some nanoamperes are needed.^7,49^ Here, we were able to demonstrate currents above 3 nA through atmospheric nitrogen and air with an emission area of only 300 μm × 300 μm. A further reduction in the substrate current density, either by increasing the total area or reducing the total current, would result in a decrease in degradation,^25^ while still providing sufficient current for such applications. This showcases that the PPF-based PHEE is a promising candidate for miniaturized gas applications.

## Conclusion

It was demonstrated that planar hot electron emitters (PHEE) equipped with ultrathin pyrolyzed polymer films (PPF) as the gate material exhibit high transfer ratios, reaching up to (30.9 ± 0.5)%. While a batch-to-batch variation of the performance is observed, a long-term stability test of PHEEs on a 4″ wafer revealed that the devices exhibit minimal fluctuations across a single substrate, with a standard deviation of only 2.9% in transfer ratio, indicating a high degree of uniformity. The degradation of the tunnel oxide observed in this experiment – characterized by an increase in the voltage required to maintain a constant current – only slightly reduced the transfer ratio. This demonstrates the high stability of the presented PHEE. Furthermore, it was demonstrated that 3 nA could be emitted through nitrogen and air with an emission area of only 300 μm × 300 μm. It can therefore be concluded that PHEEs based on PPF gates have potential applications in gas analytics. The introduced process for gate layer deposition is highly integratable and can be readily adapted by the semiconductor industry. It consists solely of photolithography and pyrolysis of polymer films. Moreover, the entire device comprises only silicon, silicon dioxide, and carbon, thus rendering it an environmentally sustainable fabrication process. In general, PPF is a noteworthy material, offering an easily scalable and patternable process alongside high functionality. Raman spectroscopy analysis of the fabricated films demonstrated that the ultrathin PPF exhibited higher crystallinity compared to the thicker PPF used as contact electrodes. This is likely to be the result of the thickness of the material being reduced to a mere few monolayers of carbon atoms, thus reducing the probability of crystallite overlapping. The markedly high conductivity of up to 3.5 × 10^–4^ S/m, which surpasses the results of previous research on PPF by a factor of 2 at a pyrolysis temperature of 900 °C, has been achieved at layer thicknesses of only (0.98 ± 0.5) nm. This demonstrates that such ultrathin PPF sheets have significant potential as a highly functional and easily accessible 2D material. Furthermore, the simple fabrication process with high throughput and yield makes this an optimal choice for further investigation of PHEEs, including studies into temperature, gate thickness, and oxide thickness dependencies, which will elucidate the processes involved in the PHEEs. Similarly, with the PPF-based gate layer, the emitter has significant potential for optimization, which may further enhance the transfer ratio and broaden the scope of applications for it.

## Supplementary Material



## Data Availability

The data are available from the corresponding author upon reasonable request.

## Notes

Fabrication scheme of TLM structures (Figure S1); methodology and contact resistance of TLM measurement (Figures S2 and S3); methodology of XPS and Raman spectroscopy; measurement of monolayer graphene PHEE (Figure S4); long-term measurement of PHEE (Figure S5); estimation of thermal load (PDF)

## Abbreviations

PF: Polymer Film
PPF: Pyrolyzed Polymer Film
PHEE: Planar Hot Electron Emitter
SEM: Scanning Electron Microscopy
XPS: X-ray Photoelectron Spectroscopy
AFM: Atomic Force Microscopy
