# Supplementary material for: Highly Efficient Planar Hot Electron Emitters Based on Ultrathin Pyrolyzed Polymer Films
Source: ACS Appl Mater Interfaces. 2025 May 30;17(23):34637–46. doi: 10.1021/acsami.4c19809 (PMC12163920; doi:10.1021/acsami.4c19809)
Supplement: Supplementary file 1 [file am4c19809_si_001.pdf]

Supporting Information

Highly Efficient Planar Hot Electron Emitters Based on Ultrathin Pyrolyzed Polymer Films

Florian Herdl[1,‡], *Natalie Galfe*[1,‡], *Sebastian Klenk*[1], *Michael Dillig*[1], *Silke Boche*[1], *Michael Bachmann*[2], *Andreas Schels*[2], *Simon Edler*[2], *Florian Dams*[2], *Andreas Pahlke*[2], *Georg S. Duesberg*[1,*]

[1] Institute of Physics & Center for Integrated Sensor Systems (SENS), University of the Bundeswehr Munich, Neubiberg, 85579, Germany

[2] KETEK GmbH, Munich, 81737, Germany

‡ These authors contributed equally.

* Correspondence to: duesberg@unibw.de

Methods

Transfer Length Method (TLM).

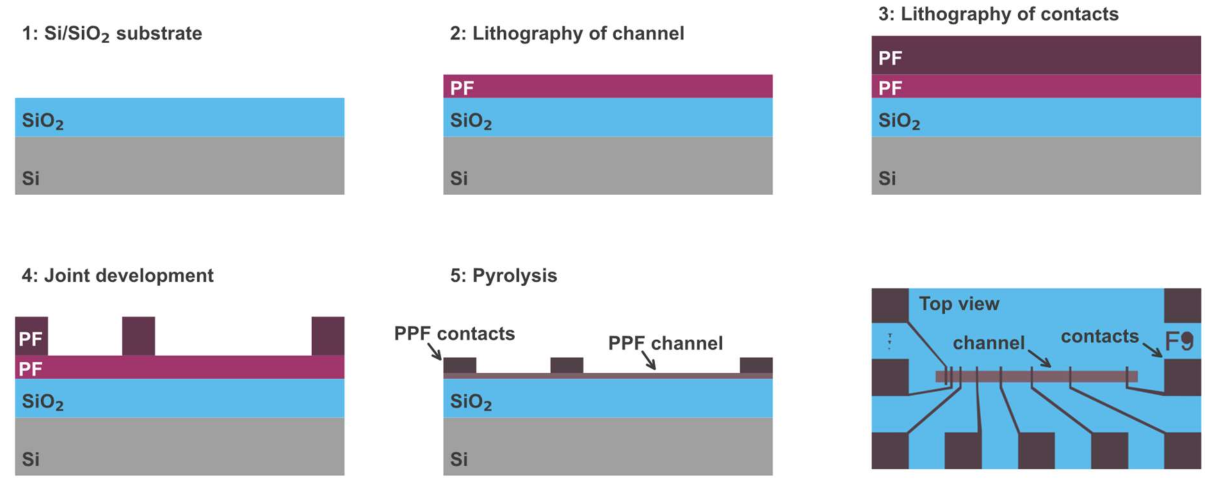

Figure S1. Fabrication scheme for the transfer length method devices. The channel and contact polymer films (PF) are defined on a Si/SiO_2 substrate (1) and patterned by lithography (2,3) and a joint development step (4). The resists are subsequently pyrolyzed (5), forming pyrolyzed polymer films (PPF). In the last image, the schematic top view of the TLM-die is shown.

TLM structures were fabricated, using the same process as described for the gate layer of the planar hot electron emitter. Figure *S*1 depicts a schematic process flow for the fabrication of a single TLM device. A total of 61 TLM dies are simultaneously fabricated on a 4" wafer. The process starts with a wet oxidized silicon wafer, the oxide thickness of which is approximately 300 nm (1). In the second step, a 5 wt% diluted polymer film (PF) is applied to the wafer, by spin coating. This is followed by exposure to UV light via lithography to define the channels (2). Before developing the channel layer, the undiluted PF is applied and patterned by lithography for the contacts (3). In a joint development step, the entire TLM structures are formed (4). In the last step, they are pyrolyzed at a low pressure of approximately 1 mbar Ar at 900 °C to form the pyrolyzed polymer film (PPF) (4).

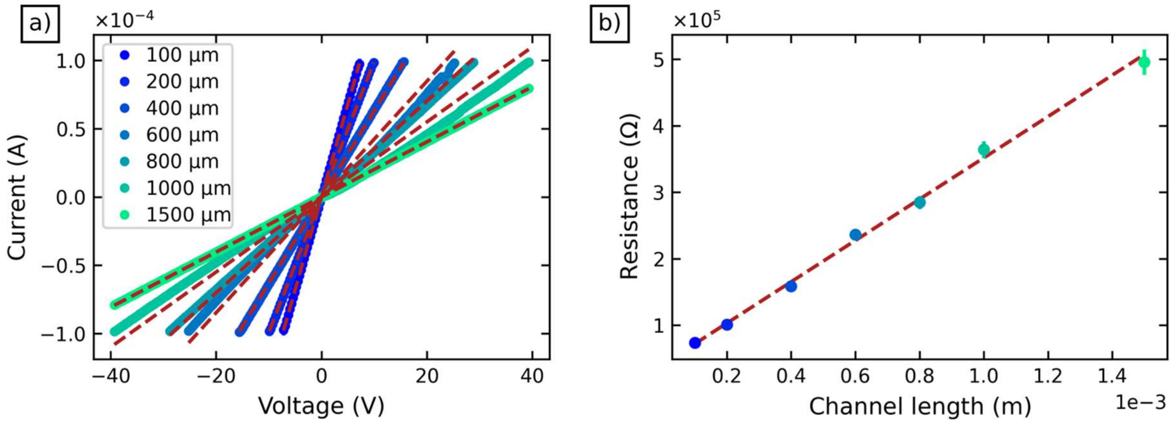

Figure S1. Exemplary evaluation of electric transport through pyrolyzed polymer films with transfer length method (centre chip). a) I-V characteristics of channels with various lengths. b) Resistance versus channel length with the linear fit in red.

Figure S2 provides an exemplary evaluation of the sheet resistance measurement for the central die (black marking in Figure 3a, main text), which exhibits the highest measured conductivity. To ensure the absence of any asymmetric effects, the IV characteristics were measured in both polarities. To monitor 61 devices with seven channel lengths on each, the entire wafer was subjected to examination using an automated probing apparatus. This could occasionally result in poor contacting, which leads to an initial shift in the linear regime. It should be noted, that a Schottky contact could also cause non-linearity at low voltages. Since in this contribution we only extract sheet resistances and to circumvent erroneous fitting, exclusively values in the linear regime at elevated voltages were considered for the linear regression. Furthermore, the negative and positive regime slopes were fitted separately. The resistance was calculated as the mean of the slopes from both polarities, along with their respective standard deviations. The linear fit illustrated in Figure S2a is the fitted curve, comprising the average slope of the two polarities, which aligns very well with the linear portion of the curves. Non-linearities were not frequently observed and only at low voltages. Additionally, the plot of resistance versus length (Figure S2b)) evinces a linear relationship, thus, this determination of the sheet resistance is deemed appropriate.

In **Figure S3**, the contact resistance distribution over the investigated 4" wafer can be seen. It shows good consistency with the measurements presented, as it resembles the resistances of the PPF leads, approximated by:

$$R_c = \frac{L}{\sigma \cdot t_l}, \tag{1}$$

with L the lead length (≈1 mm), σ the determined conductivity ($0.7 \cdot 10^4$ S/m; from manuscript and literature)[1], and t_l the lead thickness (63 nm; from manuscript) to $2.2 \cdot 10^6$ Ωµm.

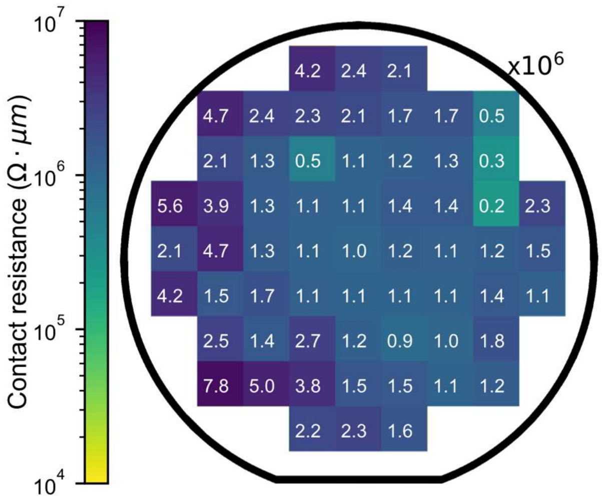

Figure S2. Distribution of the contact resistance over the investigated 4" wafer.

Raman spectroscopy. The Raman analysis was carried out with a WITec alpha 300R Raman imaging spectrometer equipped with a 532 nm laser. The integration time was set to 2.5 s with 3.8 mW of laser power and a 100x objective. To mitigate focal errors due to height differences, the TrueSurface functionality of the WITec system was used. The extracted Raman spectra of this contribution were obtained by averaging the data of the shown Raman map, from the emission and contact area, respectively.

X-ray Photoelectron Spectroscopy (XPS). For XPS measurements, a PHI VersaProbe III instrument equipped with a micro-focused monochromatic Al Kα source (1486.6 eV) and dual beam charge neutralization is used. Core level spectra are recorded with PHI SmartSoft VersaProbe software and processed with CasaXPS. Binding energies are referenced to the silicon signal of silicon oxide at 103.3 eV. The background is approximated using a Shirley background. The components are fitted as follows: The sp^2 component is fitted using a Doniach-Sunjic peak shape with (0.1,1100,52) parameters. This approximates the asymmetric nature of the sp^2 peak precisely and has been optimized for pristine graphene beforehand. All other components were fitted using the Lorentzian asymmetric peak shape LA(50).

Planar Hot Electron Emitter Based on Monolayer Graphene as the Gate Electrode

For comparison, a planar hot electron emitter (PHEE) with catalytically grown copper (Cu) and wet chemically transferred monolayer graphene was fabricated. Figure S4 shows an IV-characteristic of the PHEE with a monolayer graphene as the gate electrode. With a tunneling oxide thickness of 13 nm, the device can be compared to the PPF device presented herein. While the emitter exhibits similar transfer

ratios compared to the PPF gate layer, the yield is very low due to wrinkles and cracks that form during the transfer. Furthermore, it should be noted that the process is not easily scalable to a wafer level, and the fabrication is comparably cumbersome. Since our suggested PPF layer shows similar or higher transfer ratios to the monolayer graphene, this indicates that at such low thicknesses of the gate layer (≈1 nm) the transfer ratio might not only be limited by the gate but also by the oxide or impurities at the interface, as already anticipated by Murakami *et al.*[2] However, to fully address the complex underlying mechanism, further studies are planned.

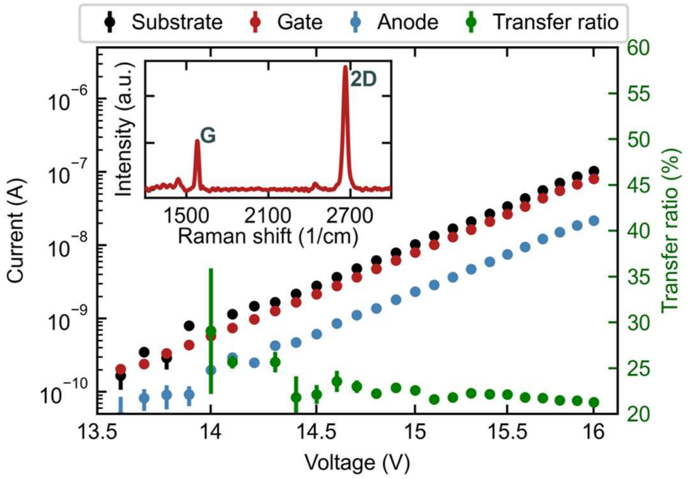

Figure S4. I-V characteristic in Millikan-Lauritsen representation of a planar hot electron emitter with a monolayer graphene as gate.

Long-term Measurement of the Planar Hot Electron Emitter (PHEE)

A lifetime measurement is shown in **Figure S5**. The observations align well with the picture described in the manuscript, as the gate voltage increases over the course of the measurement, while the anode current, and therefore the transfer ratio, decreases slightly. It seems that the reduction rate of the transfer ratio over time is nearly the same. This is probably caused by the introduction of new traps during operation, which act as additional scattering centers within the oxide. It should also be noted that different conduction mechanisms could be introduced during operation, such as Poole-Frenkel conduction[3], lowering the transfer ratio. Despite the large shift in gate voltage observed, the reduction in transfer ratio is rather low over the course of their lifetime and still show high values. The deduced charge to breakdown (Q_{BD}) is 0.43 C/cm^2. This value is low compared to MOS devices with similar oxide thicknesses (e.g. 10-20 nm; Q_{BD} ≈10 C/cm^2) [4].

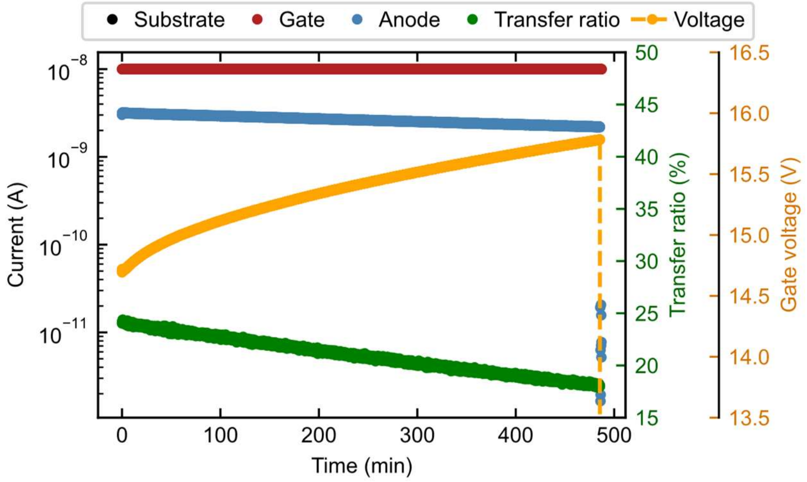

Figure S5. Lifetime measurements of the presented planar hot electron emitter.

The origin of the breakdown is often associated with a critical number of traps introduced during operation.[5] They form a conductive path through the oxide, bypassing the necessary tunneling process into the oxide conduction band, ultimately leading to a sudden stop of the observable emission current. Additional measurements on MOS devices with an aluminum gate electrode were performed, which show similarly low Q_{BD} values. This indicates that the low Q_{BD} does not originate from degradation of the oxide, e.g. during heating in the pyrolysis step, but rather from the higher initial defect density in the oxide. Further optimization on the oxide layer material, thickness, quality, and its correlations with the transfer ratio are planned in the future.

To assess the impact of heating within the stack and the possibility of thermal runaway, the following estimations are made. The thermal heating of the thin oxide can be roughly estimated by the maximum power dissipated within it and compared to the thermal conductance of the oxide layer:

$$jV = k\frac{dT}{dx}\ , \tag{2}$$

whereby j is the maximum current density of the shown measurements (j=2.2 10^{-5} A/cm^2) at an emission area of 300 µm x 300µm and V the voltage (16 V) applied over the oxide. k is the thermal conductivity of the oxide (1.15 W/(m K))[6] and dT/dx is the Temperature gradient. The maximum dissipated power is therefore 0.35 mW/cm^2, which results in a temperature gradient of 3 $\cdot 10^{-9}$ K/nm. Thus, the heat dissipated in the oxide layer can be neglected.

For the gate layer, a worst-case estimation can be made by considering the highest current density. For simplification, the emission area can be approximated as a circle with the radius r. The current density depending on the distance from the middle of the emission area is stated as:

$$j_{gate}(r) = \frac{jA}{Ct} = \frac{jr}{2t}, \tag{3}$$

with j_{gate} the current density within the gate layer, j the current density through the oxide, A and C the area and circumference of the emission site, respectively, and t the thickness of the gate layer (1 nm). With this, the highest current density at the outer most circumference (corners of the emission sites; $r = \sqrt{2} \cdot$

$150\ \mu m$) can be calculated to be 2.3 A/cm^2. The maximum dissipated power per unit area (I) at the outer boundary can then be calculated by the general form:

$$I = \frac{1}{\sigma} \cdot j_{gate}^2 \cdot t, \tag{4}$$

with σ the conductivity ($0.7 \cdot 10^4$ S/m; from the manuscript). This again shows a negligible value of 755 mW/cm^2 which can easily be dissipated through the oxide layer. It should also be noted that additional heat conduction paths are available, like along the pyrolyzed polymer film and radiation to the surroundings. For the current densities described herein, which are already sufficient for application in gas environments, heating of the oxide and gate layer, as well as thermal runaway, is not an issue. However, for high current density applications (j > 10 A/cm^2) this needs to be considered carefully and, if necessary, must be adjusted by, e.g., the geometry of the emission area.

References

(1) Schreiber, M.; Lutz, T.; Keeley, G. P.; Kumar, S.; Boese, M.; Krishnamurthy, S.; Duesberg, G. S. Transparent Ultrathin Conducting Carbon Films. *Appl. Surf. Sci.* **2010**, *256* (21), 6186–6190. https://doi.org/10.1016/j.apsusc.2010.03.138.

(2) Murakami, K.; Adachi, M.; Miyaji, J.; Furuya, R.; Nagao, M.; Yamada, Y.; Neo, Y.; Takao, Y.; Sasaki, M.; Mimura, H. Mechanism of Highly Efficient Electron Emission from a Graphene/Oxide/Semiconductor Structure. *ACS Appl. Electron. Mater.* **2020**, *2* (7), 2265–2273. https://doi.org/10.1021/acsaelm.0c00449.

(3) Murakami, K.; Rommel, M.; Yanev, V.; Erlbacher, T.; Bauer, A. J.; Frey, L. A Highly Sensitive Evaluation Method for the Determination of Different Current Conduction Mechanisms through Dielectric Layers. *J. Appl. Phys.* **2011**, *110* (5). https://doi.org/10.1063/1.3631088.

(4) Park, Y. B.; Schroder, D. K. Degradation of Thin Tunnel Gate Oxide under Constant Fowler-Nordheim Current Stress for a Flash EEPROM. *IEEE Trans. Electron Devices* **1998**, *45* (6), 1361–1368. https://doi.org/10.1109/16.678579.

(5) Palumbo, F.; Wen, C.; Lombardo, S.; Pazos, S.; Aguirre, F.; Eizenberg, M.; Hui, F.; Lanza, M. A Review on Dielectric Breakdown in Thin Dielectrics: Silicon Dioxide, High-k, and Layered Dielectrics. *Adv. Funct. Mater.* **2020**, *30* (18). https://doi.org/10.1002/adfm.201900657.

(6) Zhu, W.; Zheng, G.; Cao, S.; He, H. Thermal Conductivity of Amorphous SiO2 Thin Film: A Molecular Dynamics Study. *Sci. Rep.* **2018**, *8* (1). https://doi.org/10.1038/s41598-018-28925-6.
